# Are Natural-Based Composites Sustainable?

**DOI:** 10.3390/polym13142326

**Published:** 2021-07-15

**Authors:** Luis Suárez, Jessica Castellano, Sara Díaz, Abbas Tcharkhtchi, Zaida Ortega

**Affiliations:** 1 Departamento de Ingeniería de Procesos, Universidad de Las Palmas de Gran Canaria, 35017 Las Palmas, Spain; luis.suarez@ulpgc.es (L.S.); sara.diazguzman@ulpgc.es (S.D.); 2 Departamento de Ingeniería Mecánica, Universidad de Las Palmas de Gran Canaria, 35017 Las Palmas, Spain; jessica.castellano@ulpgc.es; 3 Arts et Métiers Institute of Technology, CNRS, CNAM, PIMM, HESAM University, 75013 Paris, France; abbas.tcharkhtchi@ensam.eu

**Keywords:** polymer composites, sustainability, recyclability, biodegradation, wastes, life cycle assessment, sustainable development goals

## Abstract

This paper assesses the aspects related to sustainability of polymer composites, focusing on the two main components of a composite, the matrix and the reinforcement/filler. Most studies analyzed deals with the assessment of the composite performance, but not much attention has been paid to the life cycle assessment (LCA), biodegradation or recyclability of these materials, even in those papers containing the terms “sustainable” (or its derivate words), “green” or “eco”. Many papers claim about the sustainable or renewable character of natural fiber composites, although, again, analysis about recyclability, biodegradation or carbon footprint determination of these materials have not been studied in detail. More studies focusing on the assessment of these composites are needed in order to clarify their potential environmental benefits when compared to other types of composites, which include compounds not obtained from biological resources. LCA methodology has only been applied to some case studies, finding enhanced environmental behavior for natural fiber composites when compared to synthetic ones, also showing the potential benefits of using recycled carbon or glass fibers. Biodegradable composites are considered of lesser interest to recyclable ones, as they allow for a higher profitability of the resources. Finally, it is interesting to highlight the enormous potential of waste as raw material for composite production, both for the matrix and the filler/reinforcement; these have two main benefits: no resources are used for their growth (in the case of biological materials), and fewer residues need to be disposed.

## 1. Introduction

Sustainable Development Goals (SDG) were adopted in 2015 by all members of the United Nations, as a way to implement policies to achieve a more respectful, peaceful and fair society. They consist of a set of 17 goals to be accomplished by 2030, covering several aspects to improve people lives and protect the planet [1], from zero hunger to gender equality or sustainable cities and communities.

From the 17 objectives mentioned, this paper places a special emphasis on the following ones, some of their specific goals also being detailed below:

- 8. Decent work and economic growth, including the increase in global resource efficiency in consumption and production or to higher levels of productivity through diversification.
- 9. Industry, innovation and infrastructure: sustainable industrialization, competitive economy and enhanced scientific research and upgraded technological capabilities.
- 11. Sustainable cities and communities: sustainable urbanization, protecting natural and cultural heritage or increase in resource efficiency.
- 12. Responsible consumption and production: implementation of responsible consumption patterns, environmentally sound management of waste, reduction of waste generation, etc.
- 13. Climate action: reduction of carbon dioxide and other greenhouse gas emissions.

It is clear that sustainability is nowadays of great concern for citizens, and many products or services can be found with the “eco”, “green”, “bio” or “environmentally friendly” labels, among others, as a marketing strategy to attract consumer attention. However, not all these labels are really referring to an actual improvement of the ecological footprint of the product or service, but are just a claim, in many cases unjustified; this is what is called “greenwashing” [2]. Some of the most well-known and recognized eco-labels are the EU-ecolabel, the Nordic swan or the blue angel (for countries) or the energy star or OEKO-tex labels [3].

On the other hand, polymers (or more specifically, plastics) are socially targeted as being responsible for many of the environmental problems found nowadays, without taking into account the impressive advantages that these materials provide compared to other ones, such as metals or glass. Plastics really constitute an environmental problem, due to their long lifetime and their abuse in products with short period of life. For this reason, several countries in the world and the EU have established regulations with increased limitations for the use of single use plastic products; in the EU, this regulation comes into force in July 2021 [4].

This paper deals with several concepts, which are explained in Section 3, such as sustainability, design and eco-design, biodegradability, recyclability, biorefineries and their relationships with polymer composites, with the aim of obtaining a picture of the state of polymer composites sustainability and in order to determine the current trends in research in this issue. More specifically, the paper focuses on the use of natural fibers or matrices to obtain composites, and their relationship with sustainability.

## 2. Materials and Methods

A systematic bibliographic search has been performed in order to determine the relevance of this topic within the literature. The combinations of keywords used and the number of documents recovered from four databases are summarized in Table 1:

This last search on Google Scholar provides around 10,200 results. As the last combination includes all the elements of interest (not all search engines support the use of Boolean operators), the analysis of the publications’ data (year, journal, cites) will be performed on this last option.

**Figure 1:**
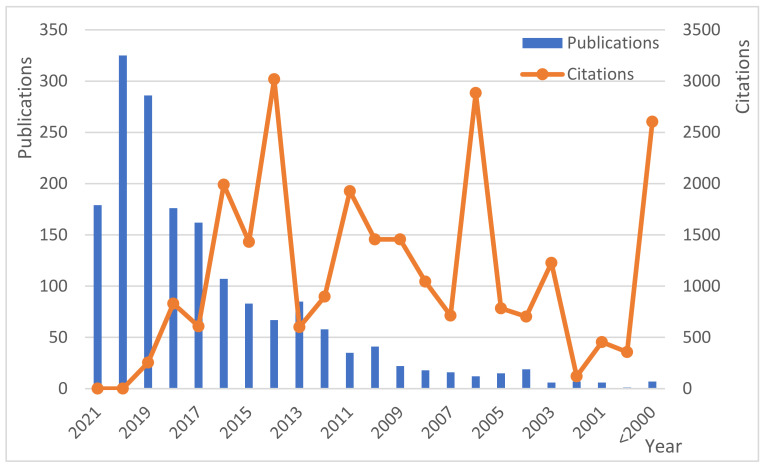
Publications and paper citations since 2000 in Scopus.

A clear upward trend can be observed in the number of publications within the last two decades, starting with only a couple of yearly publications and reaching over 300 in the last year (Figure 1). The next figure also shows the number of citations of papers published each year (that is, how many times papers published each year have been cited along the period studied; only 100 more cited papers are included). No trend is observed here, as expected; however, papers published in the years with fewer publications have had many more citations than those published in years with a higher number of publications on this topic.

Regarding areas of publications, materials science and engineering account for more than 50% of the publications in Scopus (Table 2), similar to that in Wiley. Materials (polymers) science is the most important area in which papers dealing with polymer composites and sustainability are published. It is interesting to note that only 6% of papers are found in the area of environmental science in Scopus and lower in other publications (3.2% in Wiley, considering Life sciences, Earth science and environmental studies). Moreover, it is interesting that biochemistry and medical sciences are of relative importance.

**Figure 2:**
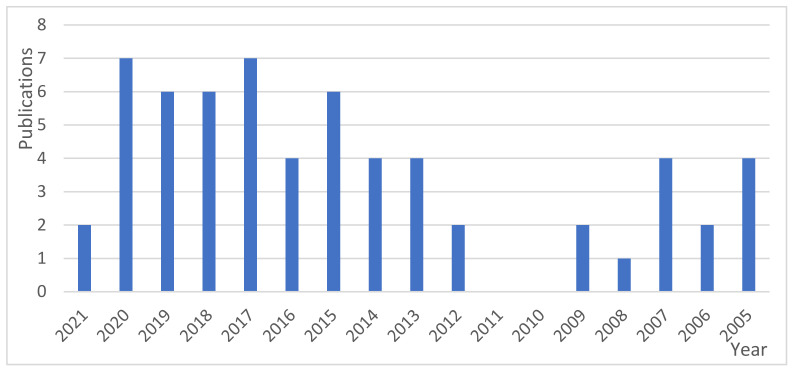
Publications in the last few years for “polymer composites” + biorefineries.

A more specific search in databases can be done, combining the terms “polymer composite” and “biorefinery”, linking both sectors as a way to take into account agro-industrial processes (wastes and side streams) and their possible use for the production of matrices or fillers. Fifty nine papers are found in Scopus with this combination, most of them published in the last 5 years and none before 2005 (Figure 2).

**Figure 3:**
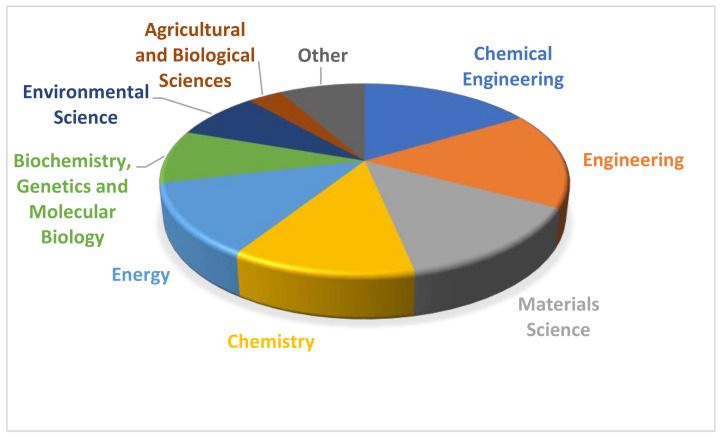
Areas of publication of papers dealing with polymer composites and biorefineries.

On this occasion, papers are included mostly in Chemical Engineering, Engineering and Materials Science, while Environmental Science is not as low as was previously commented (Figure 3).

From the over 260 papers recovered from the literature search, the terms listed below were analyzed, obtaining the relative importance summarized in Table 3:

## 3. Concepts

### 3.1. Sustainability

**Figure 4:**
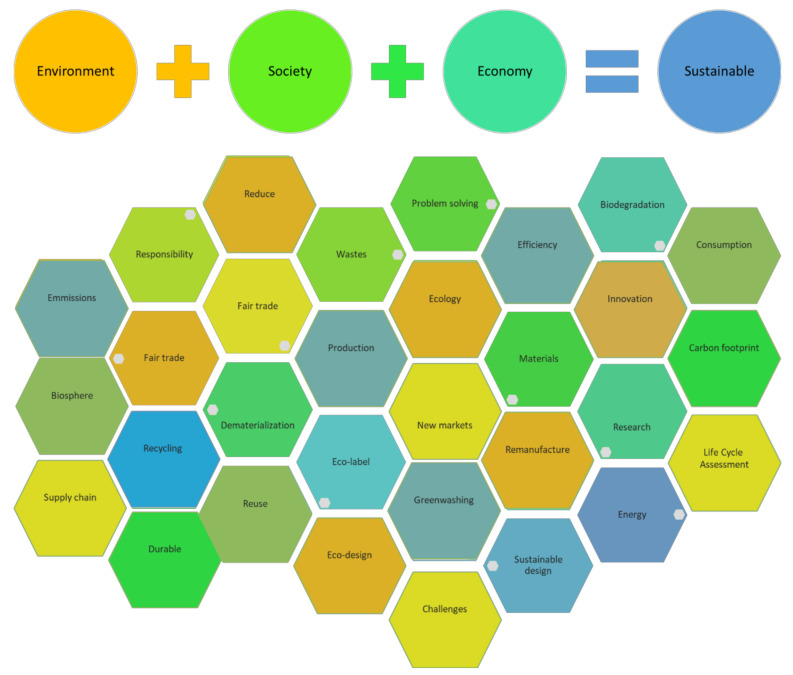
Sustainability and some associated concepts.

Sustainability, according to Brundlant’s report, is defined as: “Meeting the needs of the present without compromising the ability of future generations to meet their own needs” [5]. Sustainability then seeks a balanced use of environmental, social and economic resources, that is, to provide benefits and profit for people, planet and business with an equitable use of resources, the so-called “triple bottom line” [2] (Figure 4).

The development of sustainability principles has allowed the beginning of a shift from a cradle-to-grave model towards a cradle-to-cradle one; the first one consists of the extraction of valuable materials from nature to obtain products, which are later disposed in a landfill. This strategy dominates modern manufacturing, as it is often cheaper to buy a new product than to repair the original item [6]. The cradle-to-cradle movement aims at transforming materials considered as waste into raw materials, approaching the eco-efficiency concept; that is, “doing more with less” [7].

### 3.2. Design and Eco-Design

“Green design” or “eco-design” is not a new concept, even if it has gained relevance in the last few years. A green design is usually defined as the design process in which the focus is placed on the environmental impacts of the product, while eco-design considers the environmental impacts associated with a product during its whole lifecycle. On the other hand, sustainable product design deals with the reduction of the environmental impact of a product, such as eco-design, but it also includes economic, ethical and social factors within the design process [8].

### 3.3. Degradability, Biodegradability, Oxodegradability and Fragmentation

It is important to set the specific vocabulary to use when referring to degradation in the plastics sector. Standard CEN/TR 15351 IN [9] is a guide defining these technical words. It should be noted that the breakage of a polymer material in fragments does not necessarily correspond to the decomposition of its macromolecules. The ultimate degradation state corresponds to the formation of biomass, carbon dioxide (or methane in the case of anaerobic biodegradation) and water.

Degradation is referred to in this standard as the alteration of macromolecules by chemical dissociation of the main chain; this process is often associated with the reduction in properties as a consequence of the lower molecular weight. On the other hand, biodegradation consists of this alteration, but is caused by any type of cells; this means that the degradation of a polymer by means of enzymes isolated in laboratory conditions cannot be considered as biodegradation.

The term fragmentation makes reference to the degradation of a material into smaller pieces, produced by any mechanism.

Finally, oxobiodegradation consists of a degradation process combining an oxidative process and a biological one, happening in a simultaneous or successive manner.

### 3.4. Recyclability

For the Environmental Protection Agency in the United States, recycling is the process of collecting and processing materials to turn them into new products, preventing them to be thrown away as a residue [10]. The EU’s hierarchy of residues establishes recycling as the third preferrable option, reduction and reuse being the first ones. The EU waste framework directive [11] establishes that reuse and recycling of municipal wastes should reach at least, 55%, 60% and 65% in 2025, 2030 and 2035, respectively.

McDonough and Braungart [6] suggest that recycling systems do not allow for an efficient use of materials, as it is usual that these have lower properties than raw ones.

It is important to remember here that, as most parts of public campaigns emphasize recycling, that reduction is the most effective tool to avoid pollution and resource depletion.

## 4. Polymer Composites

**Figure 5:**
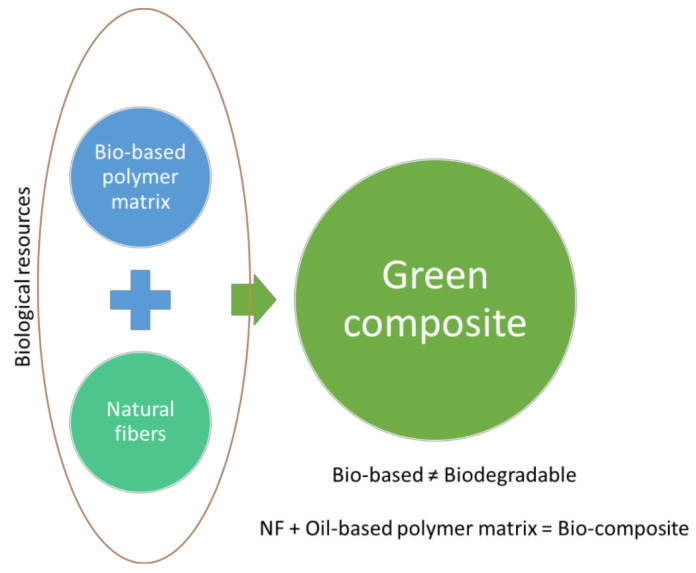
Bio-composites, bio-based composites and green composites.

A polymer composite is defined in this paper as any composite material in which a matrix consists of a polymer, a thermoset or a thermoplastic, the other material being of a different nature (being a polymer or not). In this section several alternatives to increase polymer composites sustainability are addressed, focusing on the use of alternative raw materials with a better environmental behavior, to obtain a recyclable or biodegradable composite, also considering the use of wastes or by-products as a source of such materials, either for the matrix or the filler/reinforcement, as a way to increase resource efficiency. The principles of green chemistry highlight the use of renewable feedstocks which are a needed component to avoid resource consumption and depletion [12]. Therefore, biobased fibers and matrices are seen as an alternative to obtain green polymer composites, but is their biobased origin enough to state that they are more sustainable than those containing synthetic/oil-based compounds? Anastas and Kirchoff [12] list the procurement of degradable, recyclable or reusable innocuous polymers as research challenges to focus on. Figure 5 represents the relationships between natural fibers and bio-based and green composites.

The noticeable increase in environmental consciousness of consumers indicates, together with the new regulations and the rise in fossil fuel cost, makes it likely that the use of composite materials, which include natural fibers or naturally derived matrices, will continue to grow, placing an important weight on the biobased economy [13]. There is an important number of works dealing with sustainability and composites, but which focus on other types of composites, such as concrete [14,15,16]; these are not included in this study.

This section is divided into four parts; in the first one, a brief review of the different matrices obtained from biobased sources is shown, including the relationship of polymer industry and the biorefinery concept. In the second one, several papers dealing with the use of natural materials commonly used as a reinforcement of filler to obtain polymer composites are described. This section shows an overview of this topic, as it is widely covered in the literature. Finally, the third section focuses on life cycle assessment of composites from a natural origin and the fourth one with their recycling possibilities and their biodegradation behavior.

### 4.1. Matrices

The biorefinery concept focuses on the sustainable conversion of biomass into a wide range of products, materials, and energy [17]; polymer matrices and fibers or fillers are some of such products that can be obtained from a biorefinery.

Soy bean oil can be used to obtain a thermoset polymer, which is water degradable and shows good mechanical properties, even allowing the recycling of the polymer via ester bond hydrolysis [18]. Polyamide 11 can be obtained from castor oil, being commercialized under the name Rilsan [19].

Polyhydroxyalkanoates (PHA) are a promising alternative to conventional polymers, as they are fully biodegradable and are also biobased (obtained from bacteria), and show a wide range of properties, similar to polyolefins, depending on their molecular weight [17]. In this sense, Corrado [17] proposes the use of an inulin-—rich biomass (from plants roots) and a microbial fermentation, obtaining high polymer yields, while Kumar also obtained polyhydroxybutirate (PHB) from vegetal wastes, and reinforce the matrix with cellulose also coming from wastes (sugarcane bagasse in this case) [20]. Rigid polyurethane foams can also be synthesized from waste vegetable oils, through a transesterification reaction [21,22].

Zhang [23] compiles several papers studying the potential of algae for bioplastics obtaining, concluding that despite its high potential, little information on optimal strains and culturing is available. Algae can produce lipids, carbohydrates and proteins, from which bioplastics, such as alginate, carrageenan or PHA, can be obtained, although further improvements in technology are needed in order to have a commercially viable product, also following a biorefinery approach. Algae are considered a promising feedstock due to their fast growth rate and CO_2_ absorption capacity, and be used also as reinforcement of polymeric matrices, in the same way as other natural materials.

The transformation of agro-materials inside the biorefinery’s context is also discussed in the literature, starch being a relatively wide-used material for a biodegradable low lifespan product, which can be processed via conventional twin screw extrusion [24]. De et al. [25] have proposed the use of starch combined with cellulose nanocrystals, both from agro-industrial residues, as a sustainable way to obtain a biodegradable composite material. Gil-Ramírez and collaborators [26] propose the sequential extraction of saponins, xylan and purified cellulose as an approach of integrated biorefinery to valorize agro-industrial residues (quinoa stalks in this case), obtaining highly purified cellulose at the micro-scale, suitable to be used for composite production. Kaur has also recently analyzed the role of biorefineries in obtaining polymers (both thermoset and thermoplastic) from biomass residues, stating that these are of key importance for the development of small industries devoted to the obtaining of polymers (such as poly(furfuryl)) alcohol and PFA, which is a biobased polymer with flame retardant characteristics [27], but also biochar, sweeteners for the food industry, adhesives or cellulose nanofibrils with application in composites with antioxidant, antimicrobial or superabsorbent properties [28]. Peng [29] proposes the use of hemicellulose as a natural renewable raw material for the production of ethylene-vinyl alcohol, polyvinylidene chloride or hydrogel networks, which are fully biodegradable; however, hemicellulose needs to be chemically modified to reduce hydroxyl and carboxyl free groups and increase the polymer chain stability. “Lignin-first” biorefinery has been proposed by Carpita and McCann [30] in order to increase the value of lignin-derived compounds, removing first aromatic compounds, and then processing cellulose and other carbohydrates. This approach allows valorizing lignin, which constitutes up to 40% of plants, and which is mainly devoted to energy gain by transforming the biomass into epoxy resins [31], and also to providing cellulose nanofibers and nanocrystals [30], with great interest also in the composite industry. Lignin-based bioplastics for agricultural applications and food packaging have been compiled [32], also showing its potential as reinforcement. However, all papers consulted in the transformation of lignin into polymer matrices agree in the lack of economic feasibility of this process nowadays [33].

### 4.2. Fillers and Reinforcements

Natural fibers (NF) are one of the most-used green fillers or reinforcements into polymer matrices [34]; in fact, over 1000 types of natural fibers have been used for composite production [35]. For several authors [36,37,38], the use of natural fibers allow an improvement in mechanical properties and reduce weight, but are also interesting as acoustic and thermal insulators. Besides, vegetal fibers are a renewable material and can be considered neutral in carbon, as the emissions, due to processing or even burning, are compensated with the CO_2_ absorbed by the plants to grow. Many authors consider cellulose and its derivates (including NFs) as eco-friendly materials [39,40], which allows obtaining green composite materials [41,42,43]. Some authors have also proposed the use of animal fibers, such as silk or feathers, as reinforcement of polymeric matrices [44,45]. However, NF show a poor compatibility with most polymer matrices, and several treatments are proposed to increase the affinity between both materials; the impact of such treatments may be considered when assessing the overall sustainability of the composite. Some authors propose the use of enzymes as a sustainable approach for fiber degumming [46]; another option is the introduction of biobased coupling agents, such as isocianates coming from cashew nut or naturally occurring polysaccharides, such as chitin or chitosan [47].

Adekomaya refers to the use of natural fibers in what is called “green composites” [43] as a strategy to improve the environmental performance of vehicles in two different ways: the incorporation of a renewable material, but also by the fuel consumption reduction aroused by the lower weight of the vehicle due to the use of these fibers. This paper also compiles some examples of vehicles using natural fiber composites in Toyota, Ford or Faurecia, and makes for brilliant future prospectives for these composites in the automotive industry.

On the other hand, Bismarck highlights the potential of NF in different sectors, including the automotive industry, to enhance agricultural activity, but warns that trading conditions should be reviewed to ensure fair living conditions for farmers [48]. This is an important issue to consider, as many of the NFs found on the market and used within the composite industry come from economically disadvantaged countries, and, as explained above, sustainable development also needs social and economic benefits to be truly considered as such; besides, it is of key importance to use fibers that do not displace food production. Cellulose is considered as a viable source to produce nanofibers available to substitute petroleum-based polymers, especially in spinning processes, as cellulose and its derivatives are easily processed [49].

Natural fiber composites have been widely studied in the last few years, with a wide variety of fibers being used, from cotton to jute, hemp, flax abaca, banana, to list only a few vegetable ones [19,36,50,51,52,53]. The main benefits claimed for NF are its low density and the high specific properties, apart from its renewable character. Many literature reviews can be found making these claims. Müssig and collaborators, through the MultiHemp project, proposes the concept of an integrated biorefinery to produce long hemp fibers for textile applications, short fibers for composites production, shives for construction purposes and the recovery of other compounds (oils, proteins or waxes), thus increase the efficiency of the current fiber extraction processes most commonly used in Europe [54].

Not only are natural fibers and wood interesting in the composite sector, but Al Mamun et al. [55] explain that lignin (one of the NF constituents) is a perfect candidate to obtain composites, mainly due to its abundance (as for NF), but with the extra benefits of being a by-product in pulping and biorefinery industries and showing antioxidant properties, due to its aromatic nature. Cotana states that residual lignin from bioethanol production using *Arundo donax* as raw material is suitable to be used as filler in composites, as composition and thermal degradation profiles look favorable [56]. Lignin has been successfully used for the reinforcement of several matrices (PP, PE, PLA or PVA among others) [34,57,58], with applications in energy storage devices, adhesives or biosensing [32]. Moreover, Vaidya [59] has used lignin from a saccharification process in a biorefinery as a reinforcement of a PHB matrix, obtaining a composite suitable to be 3D-printed, reducing the warpage observed for neat PHB printed parts. Moreover, lignin has also been investigated as a sustainable precursor to obtain carbon fibers [60]. Talc, mica or calcium carbonate have also been used as fillers of biobased biodegradable matrices (such as polybutylene succinate, PBS, or polybutylene adipate terephthalate, PBAT), increasing their mechanical properties and reaching values similar to polyolefins, while keeping their biodegradable character [57].

Waste materials can also be incorporated into polymer matrices to obtain composites with interesting properties. For example, Bakshi and collaborators [58] have obtained a lightweight material with a low thermal conductivity, which is suitable to be used in civil applications, by adding marble waste particulates into a polypropylene matrix in up to 80% weight. The incorporation of marble particles into the polypropylene matrix led to the increase of tensile and flexural strength. *Areca* nut [42], banana crop residues [61], grains from distillery [62], sugarcane bagasse [63], sunflower husk wastes [64], pineapple leaves [65] or woof flour [66] have been proposed in the literature as a way to obtain sustainable composites from agro-industrial wastes, to just cite a few examples.

### 4.3. Life Cycle Assessment (LCA) of Composites

Holbery and Houston [67] do not perform an environmental assessment of glass and natural fibers composites, but perform a comparison of energy costs to obtain a flax fiber mat (9.6 MJ/kg) versus a glass fiber mat (54.7 MJ/kg). A similar study was performed by Joshi, finding that energy needed for hemp fiber production is only 10% of that needed for glass fiber production [68].

In fact, a very limited bibliography is found dealing with the performance of the environmental evaluations of polymer composites. One of them is the research conducted by Oliver-Ortega and colleagues [19], which aims at performing the LCA of a totally biobased composite made of PA11 reinforced with wood and comparing it with a conventional glass fiber reinforced PP, commonly used in the automotive sector. The PA-composite achieved similar mechanical properties to the conventional PP one, being the environmental footprint of both composites similar, due to the high energy needed in the production of the PA11 monomers.

Hermansson, Janssen and Svanstr [69] have performed a preliminary study to compare the LCA of composites containing lignin or recycled carbon fibers. They conclude that the most important factor is the shift from new carbon fibers to recycled ones or to lignin, as the energy consumption in the carbonization stage is the most important impact. However, as this paper focuses on data mining from previous literature, and very little information was found, it is still unclear where to allocate the environmental impacts of these alternatives; still, these options are not fully technologically developed, and so it cannot be stated that this is an actual solution to reduce the environmental footprint of carbon fiber composites. These authors also focus on data quality, which is not often discussed; system boundaries can greatly affect the final result of the LCA, and so these results should be considered as preliminary. The study of Oliveux et al. confirms the hypothesis of Hermansson: carbon fibers can be recycled and these fibers have a better environmental behavior than virgin ones, thanks to the great amount of energy saved in carbon fiber production [70]; these authors have not found significant differences in the replacement of virgin glass fibers with recycled carbon fibers (for same mechanical properties of the composite). Gharde points that landfilling or incineration of carbon fiber plastic composites are cheaper than recycling, although the environmental benefits are higher for their recycling [71].

The LCA study of transport pallets made of PP reinforced with reed fiber or carbon fiber (with the amount of fiber needed to have similar mechanical properties: 53% reed vs 42% glass) shows a significantly lower environmental impact for reed composites, except for nitrates and phosphate emissions to water, which is associated with reed cultivation [72]. Pervaiz and collaborators have performed a similar study in the automotive sector, in this case replacing a PP part containing 30% glass fiber by the same PP with 65% of hemp fibers, obtaining a reduction of around 50,000 MJ per ton of composite. If the use of a vehicle is considered, further reductions in carbon dioxide emissions can be achieved, due to a 21% reduction of the weight of the part [73].

Zhang warns about the possible threat relating to the use of bioplastics (such as PLA or PHA), which are derived from an agricultural plant, and put the focus on using biobased materials that are different to food crops [23]. Zini and Scandola highlight the potential of agro-industrial wastes to further enhance the environmental behavior of natural fibers in the composite industry, as the negative impact associated to water and phytosanitary products (resulting in the release of nitrate and phosphates to the medium) would then be reduced [74]. In this sense, water hyacinth waste (coming from control campaigns, as it is an invasive species) shows a lower environmental impact than other NF-composites [75], also showing 6.5 less potential warming impact than carbon fiber composites. The reduction of water hyacinth compared to other natural fibers (jute and hemp) is mainly due to the amount of water needed in the growth of the crop, which is not considered for water hyacinth, as it constitutes a residue. The authors of this study have shown that most of the environmental impact of these composites is related to the matrix used (epoxy in this case). This is also the objective of the Inv2Mac project, which aims at valorizing residues from control campaigns of invasive species by using them in polymer composites. Bachman et al. have performed a review in which they have included different papers studying the LCA of NF-composites [76]; in general terms, all analyzed papers conclude that the use of natural fibers instead of synthetic ones lead to a reduction in the environmental footprint of the composite, although for some fibers the environmental benefit is not very clear. This happens for flax fibers, due to the use of agrochemicals and the emissions from processing.

### 4.4. Polymer Composites Recycling and Biodegradation

When performing the literature review about polymer composite recycling, many authors include energy valorization (fluidized bed, pyrolysis or incineration) [71] apart from the recycling to obtain a new composite that can be further reprocessed. Energy recovery is generally a preferred option, as recycling to obtain a new composite needs energy and other resources.

Vladimirov and Bica explain the multiple benefits of the mechanical recycling of glass fiber composites [77], including the simple equipment needed, avoiding the use of solvents or high energy consumption, although the main drawback continues to be the lack of uniformity on the final product obtained, due to the different types of glass fiber composites available on the market. To overcome this, glass-fiber reinforced polymers can be recycled though pyrolysis performed in a fluidized bed, recovering around 50% of the initial glass fibers, to be reintroduced into a polymer matrix to obtain a new composite. Carbon fibers can also be recovered following a similar process, although defects on the fiber surface can arise, leading to a decrease in the quality of recycled carbon fibers [76]. Different approaches have been followed to recycle carbon fibers, apart from pyrolysis; the most usual ones are chemical solvolysis, hydro-thermolysis and steam thermolysis [78]. According to Ye, this last one takes place under relatively medium temperatures (around 600 °C), and allows a transformation of the matrix into low weight hydrocarbons, CO and CO_2_, without significant damage to the fibers, that is, avoiding the downcycle (reduction in properties) of carbon fibers observed by other authors [76].

Abdullah proposes the single polymer composites (SPC) as the only alternative to really sustainable polymer-based composites [39]. These SPCs are composite materials, which are made of the same polymer as, for example, a PE matrix reinforced with PE fibers. These materials are chemically a single material, and so the recycling process is very efficient; another example is the PLA composites, made of a PLA matrix reinforced with PLA fibers, which can be recyclable and also compostable.

Some papers can be found assessing the potential recyclability of natural fiber polymer composites; a reduction in fiber size has been observed after recycling, due to the mechanical processing of the material. This reduction, or the decrease in the aspect rate of the fibers, is a factor to consider when trying to recycle plastic composites, as it greatly affects the final behavior of the composite. Viscosity is also reduced after recycling, which can be considered positive, particularly for injection molding processes, as it allows the obtaining of more precise details [79]. Even with the reduction observed in flax fiber size, plant fiber composites can be said to show stable mechanical properties after recycling as the observed reduction in fiber size mainly arises in this first recycling cycle. For example, Bourmaud and collaborators have studied a composite made of 2 mm flax fiber in a PLLA/PBS matrix after successive cycles of extrusion and injection molding [79], observing a drastic decrease in the fiber length due to processing. A different study also from Bourmaud [80] has also shown that aspect ratio is the most relevant factor on the mechanical properties of short-fiber composites, and recycling of NF-thermoplastics reduce the fiber length, but also their diameter, thus keeping the aspect ratio relatively stable. They have also found that PP composites with sisal fibers show a decrease of around 10% in tensile elastic modulus, while for hemp this shows no change after seven cycles. Gourier and collaborators have arrived at similar conclusions for PA. The results indicate a good stability and PP flax composites; that is, mechanical properties are not significantly modified due to recycling, with a decrease in flax fiber length with reprocessing cycles (seven again) [81].

For wood fiber HDPE (high density polyethylene) composites, Akesson has found that tensile and flexural strength is reduced to less than 10% after seven cycles, without significant changes in modulus [82]. On the contrary, Soccalingame et al. [83] have also been reprocessed seven times a wood flour PP composite and have not found significant variation in mechanical properties, despite the degradation observed for the fibers, which get darker and shorter with the reprocessing.

Some authors have also performed recycling tests on composites made of a biodegradable matrix, such as PLA or PBS. Bourmaud and collaborators have studied the recyclability of flax PLA/PBS composites [84], finding that mechanical properties of the composite are retained for the first three cycles, while they are reduced for further reprocessing. As this is a biodegradable material, the authors consider this material to have a good recyclability. Flax-reinforced PLA composites show the same trend [85] and also find a reduction in fiber length and in molecular weight and glass transition temperature for the polymer. These authors also suggest that this composite is suitable for recycling, as no virgin material needs to be added for processing or for keeping good mechanical properties.

Hees [86] highlights the importance of recycling to shift from the linear to a circular economy and to develop environmentally friendly plastic composites; for these authors, composites in which the matrix and the reinforcement are made of the same polymer represent an attractive feasible alternative to conventional polymers, as polyolefins are entirely recyclable. For instance, all PP-composites are light and are highly resistant to damage (high stiffness and strength, and extraordinary impact resistance), which makes them very suitable for sports equipment, transport boxes or suitcases; these composites even show self-repairability properties. For these authors, composites containing fibers of a different nature are difficult to recycle, due to the different nature of the constituents. This is not the same conclusion as other authors, who consider that natural fiber composites are recyclable [52,67]. Zabihi et al., from their point of view [87], have incorporated phosphorylated macadamia nutshells into an epoxy resin, keeping the mechanical properties of the resin, improving fire retardancy and also increasing recyclability (via partial hydrolysis of the matrix in an acid medium).

On the other hand, Hees et al. [86] compare biodegradation versus recycling, and conclude that biodegradation is less efficient than recycling, as degradation means the loss of the original raw material plus the emissions of CO_2_ or methane during the degradation. Some authors have studied the biodegradation of composite materials for specific applications, such as wastewater decontamination [88], tissue engineering applications [89] or controlled nutrient release in soils [90].

It is well known that polyolefins are not biodegradable, although some modifications can be made to the polymer in order to decrease its molecular weight by processing, thus leading to the possibility of getting attacked by microbes [41]. For Zuccarello, natural fibers only can be considered as biodegradable when introduced in a biodegradable matrix [91]. Other authors agree in stating that the use of natural fibers in conventional polymer matrices is not enough to significantly increase the ecological character of the composite [39,92]. Bassyouny, Javaid and Hasan consider that green polymeric matrices are those biodegradable materials obtained from renewable resources [35]. Zini and Scandola agree with this definition of green composite; for them, a green composite is a specific class of biocomposite, where a bio-based polymer matrix is reinforced by natural fibers [74].

Azwa and collaborators point out that, as natural fibers are degraded by UV radiation, microorganisms or even humidity, the composites are also more susceptible to being attacked; this should be considered when designing a component made of these composites, because this can reduce the performance of the material and lead to problems during the product lifetime [93]. Sahari and Sapuan compiled several research papers studying the properties of biodegradable matrices (PLA, starch, cellulose acetate butyrates, etc.) reinforced with natural fibers (wood, sisal or flax) to increase their mechanical properties, which is one of these materials’ main limitations. No comparison on biodegradability properties of the matrix versus the composites can be found in this paper [94]. Similarly, Cao et al. studied the properties of a starch composite with bagasse fibers, studying the different behaviors for untreated and NaOH-treated fibers; despite the name of the paper (“mechanical properties of biodegradable composites reinforced…”), biodegradation assays are included in the study [95].

Zykova compiled a series of papers assessing the biodegradability of polymer composites [96] and found that the introduction of natural fibers in conventional thermoplastic matrices (such as LDPE, low density polyethylene) allows an increase in the biodegradability of the composite. The content of NF in the composite is directly related to the weight loss observed after the assay; that is, the higher the fiber content, the higher biodegradation occurs. Polyethylene glycol introduced in a PE matrix also increases the biodegradation of the composite. Kirsh and Chutkina achieved similar results when adding several agricultural residues (cocoa and rice husks or beet pulp) to a PE matrix, finding that the mechanical properties were drastically reduced after composting [97]. Finally, Saha, Kumar and Kumar have studied the performance of epoxy composites containing pineapple fibers, including biodegradability assays [65]; for these tests, test bars are subjected to a bacteria suspension for 45 days, and the weight of the samples is taken as a measurement of the biodegradation extent. Results show similar conclusions to those observed in other studies, with increased the biodegradability when introducing a higher ratio of pineapple fibers.

## 5. Conclusions

The interest in biobased materials is undeniable as a way to increase the sustainability of materials. Not many papers performing an analysis of the different aspects of polymer composite sustainability are found in the literature; from the opposite point of view, comparisons and reviews concerning biobased matrices or natural fibers are accessible online.

It is interesting to note that there is a wide number of publications dealing with the use of natural fibers or natural, biodegradable or biobased matrices, which claims to be producing a more sustainable or more environmentally friendly material than those based on fuel, although very few of them have performed environmental analysis to really demonstrate this point.

Several researches focus on recycling as a strategy to maximize the materials use, rather than on biodegradability or compostability. Many papers were found claiming that they were a biodegradable composite material, without performing any biodegradability assay and just assuming that, as one of the components is of a natural origin, the final material is biodegradable, similarly to what happens for the “sustainability” labelling.

This paper has shown that there is an increasing trend in the use of natural materials for composites obtained, with a special focus on waste materials or by-products of other processes, but that further analyses are needed in order to fully understand to what extent the use of biobased matrices and fillers/reinforcements contribute to the sustainable development goals. That is, efforts are needed to characterize these materials also in terms of environmental behavior: carbon and water footprint, actual recycling possibilities and biodegradation options, as the last step.

## Figures and Tables

**Table 1:** Keywords used in the bibliographic search and number of documents recovered from several databases.

Keywords	Scopus	Science Direct	Wiley	Taylor and Francis
Polymer composites, sustainab ^1,^*	3266	8	1757	521
Polymer composites, recycle *	4124	78	2407	721
Polymer composites, degradat *	10,273	43	7094	1960
Polymer composites, biodegradat *	2238	1	694	201
Composites sustainab *, recycle *	1733	0	10,176	4984
Polymer composites, sustainab *, recycle *	558	0	745	217
Polymer composites, sustainab *	606	0	255	263
Composites, sustainab *, recycle *, biodegrade *	161	0	2997	1121
Polymer composites, sustainab *, recycle *, biodegrade *	90	0	460	134
“Polymer composites” sustainab * or recycle * or biodegrade *	2588	-- ^2^	5124	--

^1,^ * are used to include within the search all related terms (i.e., sustainable, sustainability). ^2^ These databases do not allow this search.

**Table 2:** Area of publications in Scopus and Wiley.

Subject Area in Scopus	Publications	Subject Area in Wiley	Publications
Materials science	31.8%	Polymer science and technology	22.7%
Engineering	22.8%	Materials science	19.0%
Chemistry	10.8%	Chemical and Biochemical engineering	14.2%
Chemical engineering	8.3%	Chemistry	12.0%
Environmental science	6.4%	Physics	10.9%
Physics and Astronomy	5.7%	Nanotechnology	5.5%
Biochemistry. Genetics and Molecular Biology	2.3%	Engineering	2.8%
Agricultural and Biological Sciences	1.3%	Life sciences	2.0%
Earth and Planetary Sciences	0.8%	Biomedical engineering	1.5%
Others	4.9%	Mechanical engineering	1.3%
		Medical science	1.1%
		Earth science	0.7%
		Environmental studies	0.5%
		Others	5.9%

**Table 3:** Terms and appearance in the bibliographic search.

Keyword	Number of Coincidences	% of Papers Including the Keyword
LCA	19	2.5%
biodegradation	100	13.2%
recycling	148	19.6%
eco-composite	2	0.3%
eco-friendly	40	5.3%
green-composite	42	5.6%
bio-composite	141	18.7%
sustainable	113	14.9%
natural	122	16.1%
biobased	29	3.8%

## Data Availability

Not applicable.
